# N-acetyl Cysteine Coated Gallium Particles Demonstrate High Potency against *Pseudomonas aeruginosa* PAO1

**DOI:** 10.3390/pathogens8030120

**Published:** 2019-08-01

**Authors:** Mikaeel Young, Ali Ozcan, Briana Lee, Tyler Maxwell, Thomas Andl, Parthiban Rajasekaran, Melanie J. Beazley, Laurene Tetard, Swadeshmukul Santra

**Affiliations:** 1 Burnett School of Biomedical Sciences, University of Central Florida, Orlando, FL 32816, USA; 2 NanoScience Technology Center, University of Central Florida, Orlando, FL 32816, USA; 3 Department of Chemistry, University of Central Florida, Orlando, FL 32816, USA; 4 Department of Physics, University of Central Florida, Orlando, FL 32816, USA; 5 Department of Materials Science and Engineering, University of Central Florida, Orlando, FL 32816, USA

**Keywords:** gallium, biofilm, MIC, MBC, *Pseudomonas aeruginosa* PAO1

## Abstract

Nosocomial infections pose serious health concerns with over 2 million reported annually in the United States. Many of these infections are associated with bacterial resistance to antibiotics and hence, alternative treatments are critically needed. The objective of this study was to assess the antimicrobial efficacy of a gallium (Ga)-based particle coated with N-Acetyl Cysteine (Ga-NAC) against *Pseudomonas aeruginosa* PAO1. Our studies showed the Minimum Inhibitory Concentration (MIC) of PAO1 treated with Ga-NAC was 1 µg/mL. Cytotoxicity of Ga-NAC against multiple cell lines was determined with no cytotoxicity observed up to concentrations of 2000 µg/mL (metal concentration), indicating a high therapeutic window. To elucidate potential antibacterial modes of action, Inductively Coupled Plasma—Mass Spectrometry (ICP-MS), infrared spectroscopy, and atomic force microscopy (AFM) were used. The results suggest improved Ga^3+^ interaction with PAO1 through Ga-NAC particles. No significant change in cell membrane chemistry or roughening was detected. As cell membrane integrity remained intact, the antimicrobial mode of action was linked to cellular internalization of Ga and subsequent iron metabolic disruption. Furthermore, Ga-NAC inhibited and disrupted biofilms seen with crystal violet assay and microscopy. Our findings suggest the Ga-NAC particle can potentially be used as an alternative to antibiotics for treatment of *Pseudomonas aeruginosa* infections.

## 1. Introduction

Nosocomial infections are rising and have become a major health concern with at least 2 million nosocomial infections every year and over 23,000 deaths in the United States alone [1]. *Pseudomonas aeruginosa* was categorized as a “serious” threat by the U.S. Center for Disease Control, with over 50,000 infections annually, including ~13% from multi-drug resistant strains [1]. With the rise in antimicrobial resistance (AMR), new antibiotics and alternative treatments are needed to minimize loss of life. As predicted by the World Health Organization (WHO), the annual death toll could reach 10 million by 2050 if AMR is not addressed appropriately.

Owing to the rise in antibiotic resistance, older antibiotics previously removed from use, such as Colistin, have resurfaced. Colistin is a poly-cationic peptide that reacts with components of the anionic lipopolysaccharide (LPS) in Gram-negative bacteria. It displaces Mg^2+^ and Ca^2+^, leading to loss of membrane integrity, promoting cell leakage and eventual cell death. Originally discovered in the 1940s and used from the 1950s onwards, Colistin was eventually discontinued in the 1980s after strong evidence of nephro- and neurotoxicity [2,3]. In the late 1990s and early 2000s, Colistin once again resurfaced with new guidelines including co-administration with other antibiotics for severe cases of infections [2,3]. The reemergence of Colistin underscores the need to develop new and safer antibiotics.

Gallium is a semi-metallic element that is used for medical diagnostics. The gallium-67 isotope has been used to scan for malignant and inflammatory cells [4]. Over time, Ga usage has shifted from diagnostics to direct treatment. Gallium nitrate buffered with citrate (GaN, brand name Ganite^®^, Genta, NJ, USA) was approved by the FDA for the treatment of cancer-related hypercalcemia; however, it is no longer available on the market. More recently, gallium compounds have been studied for their potential to treat numerous infections. Gallium nitrate buffered with citrate (referred to as GaN (aka gallium citrate)), gallium maltolate and gallium protoporphyrin IX have been shown to exhibit antibacterial efficacy against *Pseudomonas aeruginosa*, *Staphylococcus aureus*, *Enterococcus faecalis*, *Klebsiella pseumoniae*, *Acinetobacter baumannii*, *Enterobacter aerogenes*, and *Enterococcus faecium* [5,6,7,8,9,10,11,12,13]. Development of a gallium-based antimicrobial compound has moved into the translational stage. Gallium citrate has undergone a phase 1 clinical trial for potential treatment of cystic fibrosis lung infections [11], whereas gallium protoporhyrin IX has demonstrated efficacy when tested using in-vivo models [14].

The antibacterial ability of Ga is derived mostly from chemical mimicry to Fe^3+^ [15]. Ga^3+^ has binding affinity to target molecules similar to that of Fe^3+^ [15]. However, unlike the Fe^3+^/Fe^2+^ redox pair, Ga^3+^ does not complete a redox cycle due to the unstable Ga^2+^ species. It is reported that the lack of redox cycling of Ga^3+^ perturbs bacterial metabolism, resulting in severe stress and eventually cell death [5,11,12,15]. As with many organisms, iron plays important roles in metabolism, biofilm formation and antimicrobial resistance in *Pseudomonas aeruginosa* [16]. It has been shown that Ga can use *P.aeruginosa* Fe-associated siderophores such as pyoverdine and pyochelin to be uptaken into the cell [5,17,18].

N-acetyl-cysteine (NAC) is a derivative of the amino acid L-cysteine with thiol groups that confer antioxidant properties. It has been shown to disrupt *Pseudomonas aeruginosa* biofilms and loosen mucus in cystic fibrosis patients [19,20]. NAC has antibacterial and antibiofilm properties against *Pseudomonas aeruginosa* [19,21] as well as an excellent safety profile. NAC has been used as a surface-capping agent for nanoparticles due to its excellent biocompatibility and water solubility [22,23].

In the present study, we have developed a novel antimicrobial treatment based on Ga-NAC and evaluated its potency for treating *Pseudomonas aeruginosa* PAO1 skin infections. The mode of action of the treatment and its activity on biofilm formation were also studied.

## 2. Materials and Methods

All chemicals were reagent grade and used without any further purification. Gallium nitrate hydrate (Cat # AC212441000), N-acetyl cysteine (NAC, Cat #AAA1540922), sodium hydroxide, tryptic soy broth and agar, phosphate-buffered saline (PBS) were acquired from Fisher Scientific (Pittsburgh, PA). Deionized (DI) distilled water was obtained from a Nanopure water purifier (Barnstead, model # D11911). Bacterial studies were conducted using Falcon microtiter plates (Cat # 08-772-2C) purchased from Fisher Scientific. The *Pseudomonas aeruginosa* PAO1 (ATCC 15692) culture was purchased from the American Type Culture Collection (ATCC, Manassas, VA, USA).

## 3. Ga-NAC Synthesis and Characterization

The gallium-based particle coated with NAC was synthesized using a sol-gel technique. Gallium nitrate hydrate (3.136 g) was stirred at ~150 rpm in a conical flask with 20 mL of DI water. After complete dissolution (~15 min) of the gallium nitrate, NAC (3.81 g) was added to the stirring solution and allowed to stir for an additional 1 h. While continuing to stir, the pH of the solution was slowly raised to 7.5 with 5 M sodium hydroxide. The solution was left to stir for an additional 2 h before the volume was adjusted to 40 mL with DI water. Morphological characterization of Ga-NAC was conducted using high-resolution transmission electron microscopy (HR-TEM; FEI Tecnai F30, Hillsboro, OR, USA), while the chemical interaction between NAC and the gallium hydroxide particle was investigated with an ATR accessorized Fourier transform infrared spectroscopy (FTIR; Perkin Elmer Spectrum 100, Waltham, MA, USA). Before FTIR analysis, the Ga-NAC solution was washed three times with DI water and then centrifuged at 10,000 rpm for 5 min. The pellet was then frozen and lyophilized to produce a powder. With a scan range of 4000 cm^−1^ to 650 cm^−1^, three scans were collected for each measurement using increments of 1 cm^−1^. The size, aqueous dispersion, and surface charge of the Ga-NAC particle compared to uncoated gallium hydroxide particle were assessed using dynamic light scattering (DLS, Malvern Zetasizer Nano ZS). The structure and morphology of Ga-NAC materials were studied using TEM imaging.

## 4. Ga-NAC Antimicrobial Studies

The antimicrobial properties of the Ga-NAC were studied using an array of standard microbiological techniques including Minimum Inhibitory Concentration (MIC), Minimum Bactericidal Concentration (MBC), and a bacterial viability assay. Samples were tested against *Pseudomonas aeruginosa* PAO1 (ATCC 15692). *Pseudomonas aeruginosa* PAO1 was chosen due to its abundance in literature serving as a model strain of *P. aeruginosa* in antimicrobial susceptibility studies and biofilm studies. *P. aeruginosa* was maintained with tryptic soy agar and 1.5% broth at 37 °C. The efficacy of Ga-NAC was compared to uncoated gallium hydroxide as well as to industry standards gallium citrate and antipseudomonal antibiotic Colistin.

### 4.1. Minimum Inhibitory Concentration (MIC) and Minimum Bactericidal Concentration (MBC)

The Minimum inhibitory concentration refers to the lowest concentration that visibly inhibits bacterial growth while the minimum bactericidal concentration refers to the lowest concentration that completely kills the bacteria as confirmed by CFU. MIC evaluation experiments were carried out using broth microdilution in accordance with the guidelines of the Clinical and Laboratory Standards Institute (CLSI) [24]. A range of concentrations from 125 µg/mL active to 0.125 µg/mL were tested for all materials and controls. PAO1 was incubated with test samples for 16–20 h at 37 °C. The MBC of Ga-NAC was determined by CFU, plating the two concentrations above and below the MIC value (data not shown). Colony counts for MBC confirmation were checked after agar plates were incubated for 20–24 h at 37 °C. MIC and MBC values were determined in Mueller Hinton broth, 1.5% Tryptic Soy Broth (TSB) and deferrated (iron-low) 1.5% TSB (D-TSB). D-TSB has traditionally been used as a Fe low media to demonstrate the improved activity due to disruption of Fe-dependent metabolic processes [25,26]. Many infections occur in what is considered Fe-low environments. D-TSB was prepared by following the same instructions as standard TSB but additionally using small modifications to previous studies, adding Chelex^®^ 100 resin (Bio-rad) (15 g/L) and stirring for 16 h at 4 °C [9,25,26]. After stirring, the resin was filtered out using a 0.4 µm filter. After autoclaving, CaCl_2_ and MgSO_4_ were supplemented to D-TSB at the final concentrations of 22.5 and 11.25 μg/mL, respectively.

### 4.2. Cell Culture and Cytotoxicity Study

Human dermal fibroblasts (HDF, ATCC PCS-201-012) and murine macrophages (J774.A1, ATCC TIB-67) were obtained from the American Type Culture Collection (ATCC), cultured in high glucose Dulbecco’s Modified Eagle Medium (DMEM) and DMEM F-12, respectively. Dermal fibroblasts and murine macrophages were chosen for cytotoxicity studies to simulate in-vitro exposure in Pseudomonas aeruginosa skin infections. Both cell culture media contained L-glutamine but further supplemented with 10% fetal bovine serum (FBS) and 1% penicillin/streptomycin. Cells were incubated at 37 °C with 5% CO_2_. Cytotoxicity testing was conducted according to the guidelines of ISO 10993-5:2009 [27]. Cells were cultured in a Nunc cell culture flask until >70% confluency. Then cells were removed and seeded into a 96-well tissue culture microplate. Each well contained 200 µL of cells at a density ~2.5 × 10^5^ cell/mL. After 24 h of incubation, the cell medium was removed and adherent cells were washed with 1X PBS. In the next step, cell media supplemented with 10% per volume of treatment was added to the well. Cells were incubated for 20–24 h at 37 °C with 5% CO_2_ with treatments ranging from 2000 µg/mL to 125 µg/mL for all treatments. After incubation, media was removed and cells were washed with 1X PBS before fresh media was added along with alamar blue dye at 10 µL/100 µL. Cells were again incubated at 37 °C with 5% CO_2._ Viable cells reduce alamar blue dye (resazurin) resulting in an increase in fluorescence signal. Using a microplate reader, the fluorescence intensity of the well was measured at 590 nm while keeping the excitation wavelength fixed at 530 nm. Cells treated with 10% DMSO were used as positive controls (Data not shown). Following ISO 10993-5:2009, treatments that lowered cell viability below 70% were considered cytotoxic. All treatments were conducted in triplicate and cell viability expressed as a percent of the untreated control.

## 5. Biofilm Studies

### 5.1. Bacterial Viability in Established Biofilms after Treatment

The effect of Ga-NAC on the viability of cells within an established biofilm was tested by growing PAO1 biofilms on nunc TSP lids in 1.5% TSB at 37 °C for 48 h. The TSP lid was moved to fresh media at 24 h. After 48 h of establishment, the lid was removed from the media and washed in 1X PBS for 3 min to remove loosely-bound cells before being placed into a new microplate with media containing serially diluted Ga-NAC, Ga citrate and Colistin. The microplate was incubated for 16–20 h before the lid was removed and washed twice in 1X PBS. After washing, the lid was placed into a new plate with 1X PBS and sonicated for 25 min to knock the remaining biofilm off the TSP lid. The 1X PBS was subsequently plated and colony counts determined after 24 h.

### 5.2. Inhibition of Biofilm Formation

The assessment of biofilm formation of *P. aeruginosa* with Ga was conducted using a modified published protocol [28]. Briefly, *P. aeruginosa* was inoculated in 10 mL of 1.5% TSB at 37 °C overnight, placed on a shaker set at 150 rpm, and the media subsequently diluted 50-fold (1:50). The initial test concentrations of the compounds were serially diluted in TSB and then supplemented with standardized culture 1:1 for a final volume of 150 µL. Control wells contained TSB as a sterility control or standardized overnight culture as a growth control. To reduce possible effects due to dehydration, the wells at the periphery of the plate were inoculated with 150 µL of sterile distilled H_2_O (dH_2_O). Biofilms were grown on polystyrene peg lids (Nunc), a method shown to produce more reproducible biofilms compared to using the surfaces of the wells [28]. After placement of the peg lid, the plate was sealed with parafilm to prevent evaporation and incubated for 24 h at 37 °C, 150 rpm. After 24 h of incubation, the lid was transferred to a new plate containing 200 µL 1X PBS and placed on a shaker at 150 rpm for 3 min to remove any loosely-bound cells. After the PBS wash, the lid was transferred to a plate containing 200 µL of 0.1% (*w*/*v*) crystal violet and incubated for 15 min. After incubation, the excess of crystal violet was removed using two washing steps with 200 µL 1X PBS for 3 min. Subsequently, the lid was submerged in 200 µL of 95% ethanol for 15 min before being removed and the absorbance of the plate was read at 600 nm.

### 5.3. Treatment of Established Biofilm

The ability of Ga-NAC particles to disrupt and reduce the established PAO1 biofilms was assessed using scanning electron microscopy (SEM). Biofilms were grown on thermanox (Nunc) coverslips submerged into a 12-well microplate with 2 mL of log 5 CFU/mL PAO1 in 1.5% TSB. The plate was incubated at 37 °C and biofilms were grown for 24 h. The established biofilms were then washed with 1X PBS and incubated with fresh media containing 625 µg/mL Ga-NAC particles. The earlier bacteria viability assay demonstrated that 625 µg/mL of Ga-NAC particles were able to achieve a 1 log reduction in bacteria viability. After 48 h of treatment at 37 °C, biofilms were washed twice with 1X PBS and fixed for 2 h at 4 °C with 2.5% glutaraldehyde in 0.1 M cacodylate buffer [29]. The biofilms were subsequently dehydrated using ethanol with increasing concentration (50–100%) and dried in a desiccator. After drying, the biofilms were coated with a thin layer of gold (a few atomic layers) with a sputter coater (Quorum Technologies, East Sussex, UK) and imaged using SEM (Zeiss Ultra-55 FEG, Oberkochen, Germany).

### 5.4. Confocal Laser Scanning Microscopy (CLSM)

*P.aeruginosa* PAO1 biofilms were studied using CLSM following a published protocol [30]. Briefly, 300 µL of PAO1 standardized to 1 × 10^8^ CFU/mL from an overnight culture in TSB was added to an 8-well chambered coverglass (1.0 borosilicate glass, Nunc Lab-Tek chambered coverglass). The chamber was incubated at 37 °C for 48 h with the media being changed with fresh media at 24 h. After 48 h of incubation, the biofilms were exposed to Ga-NAC and Ga-citrate at 625 µg/mL metallic Ga (previous CFU study determined 625 µg/mL was required to obtain a 1 log reduction in viable cells) for 12–16 h at 37 °C. After treatment, biofilms were gently washed twice with 1X PBS and stained with 5 µM SYTO 9 for 20 min in the dark. After rinsing, biofilms were examined using CLSM on a Leica TCS SP5 using a 100x objective. Laser excitation was performed sequentially at 488 nm and emission was collected from 510 to 550 nm. Image stacks were collected with a step size of ~0.35 µm at 400 Hz. Multiple fields of view (*n* = 4) were imaged to observe an average effect. To obtain quantitative information, the images were analyzing using COMSTAT software [31,32]. COMSTAT analysis was used to determine the biomass and surface area of PAO1 biofilms after treatment with Ga-NAC, Ga citrate and Colistin.

## 6. Ga-NAC Particle Mode of Action

### 6.1. Bacterial Sample Preparation

Sample preparation began after 1 mL aliquot of cultured PA01 was transferred into six microcentrifuge tubes. In a 1:1 ratio, the bacteria were treated with various treatment iterations (10 µg/mL): untreated control, gallium citrate, uncoated gallium, NAC, Ga-NAC, Gentamicin, and Colistin treatments. Sample aliquots were re-incubated for 30 min at 37 °C. After 30 min, the samples aliquots with 500 μL of each treatment were pipetted into additional microcentrifuge tubes for 24 h treatment incubation. The remaining 500 μL of each treatment were washed by centrifuging at 4000 rpm for 5 min to create a pellet, discarding the supernatant, and then adding 500 μL of dH_2_O back in. Washing was repeated three times. The same process was repeated for the 24 h samples.

### 6.2. Cell Membrane Chemistry

To identify significant changes to bacterial cell membrane chemistry that would be caused by the treatments, FTIR spectra were obtained at 30 min and 24 h of treatment. The ATR crystal of the FTIR was cleaned with isopropyl alcohol. The background IR spectrum was collected with ddH_2_O before every scan. Once the background was obtained, 10 μL of the bacteria sample was pipetted onto the crystal and allowed to settle for 1 min to increase the density of bacteria at the surface of the ATR crystal surface. Measurements were acquired at a spectral resolution of 2 cm^−1^ in the range of 4000 to 650 cm^−1^ with an average of four spectra per measurement, with quintuplicates run for each sample. FTIR spectra were analyzed using OriginLab 2016 and Unscrambler V.10.4 for principal component analysis (PCA). PCA data was pretreated by applying an ATR correction, mean normalization, and then fitted to a MSC/EMSC model.

### 6.3. Cell Membrane Degradation Determined by Atomic Force Microscopy (AFM)

AFM (NanoIR AFM, Bruker, Santa Barbara, CA, USA) was used to evaluate the surface roughness of the bacterial cell membrane after the 30 min and 24 h treatments. Aliquots from the same primary samples were used for FTIR and AFM. The bacterial solution (1 μL) was pipetted onto a calcium fluoride (CaF_2_) substrate, and air dried. AFM images were acquired in contact mode with a scan rate of 1 Hz. The cantilevers used were silicon n-type probes coated with gold on both sides, with an associated free resonance frequency of ~11–19 kHz and a force constant of 0.1–0.6 N/m (SICONG, Applied NanoStructures, Inc., Mountain View, CA, USA). Images were collected in ambient conditions (~22 °C, 50% relative humidity) with a resolution of 500 by 500 pixels. Images obtained were processed in data analysis software Gwyddion [33] to extract the root mean square (RMS) surface roughness of the bacteria. Statistical significance of RMS surface roughness measurements as a function of treatment was conducted via analysis of variance (ANOVA) with a post-hoc Tukey test in OriginLab 2016 software. The probability (*p*-value) was used to determine statistical significance between treatment types using *p*-values of ≤0.05, 0.01, and 0.001.

### 6.4. Time-Dependent PAO1 Growth Curve with Fe Supplementation

A time-dependent growth curve of PAO1 treated with our gallium material was also explored. Treatment of bacteria with materials was carried out following the same procedure as for the MIC assay. The growth curve was set up in 1.5% TSB with Ga treatment. PAO1 were treated with the Ga coated particles and controls in the presence and absence of 600 µM FeCl_3_ [9]. The growth curves were acquired for 22 h at 37 °C, and absorbance was recorded at 600 nm.

### 6.5. Ga Release from NAC Coated and Uncoated Ga Particle

Ga release by the particles was measured using a dialysis method, where 10 mL of 1250 µg/mL coated and uncoated Ga particle was placed inside a 3.5 kDa Spectra/Por 3 dialysis wall tubing. The dialysis membrane was placed in 150 mL of 1X PBS containing a magnetic stir bar and set to stir at 200 rpm. At various time points (2, 6, 12, and 24 h), 2 mL aliquots were removed from the dialysate. The Ga content in the dialysate was determined using atomic absorption spectroscopy (AAS) (Perkin Elmer Analyst 400 AAS).

### 6.6. Ga-cell Association with PAO1

Association of Ga with PAO1 cells (internalized and surface-bound) was studied to observe the uptake of Ga into PAO1. PAO1 cells were sub-cultured in 1.5% TSB overnight and subsequently diluted to Log 8 CFU/mL. Cells were treated with 10 µg/mL of Ga-NAC particle and Ga citrate for 1 h at 37 °C. After incubation, cells were washed three times with 1X PBS at 6000 rpm for 5 min and then treated with 1% sodium dodecyl sulfate (SDS) and digested in trace metal grade nitric acid. Dissolved Ga was measured using a Thermo Fisher Scientific iCap Qc inductively-coupled plasma mass spectrometer (ICP-MS) with QCell technology and operated in kinetic energy discrimination (KED) mode of analysis with helium as the collision gas. Calibration, internal, and quality control standards (Inorganic Ventures) were prepared in 2% trace metal grade nitric acid (Fisher Scientific). This experiment was repeated with Ga-NAC particle treatment only at 50, 100 and 150 µg/mL to observe the effect of concentration on Ga-cell association using AAS.

## 7. Results

### 7.1. Material Characterization

**Figure 1:**
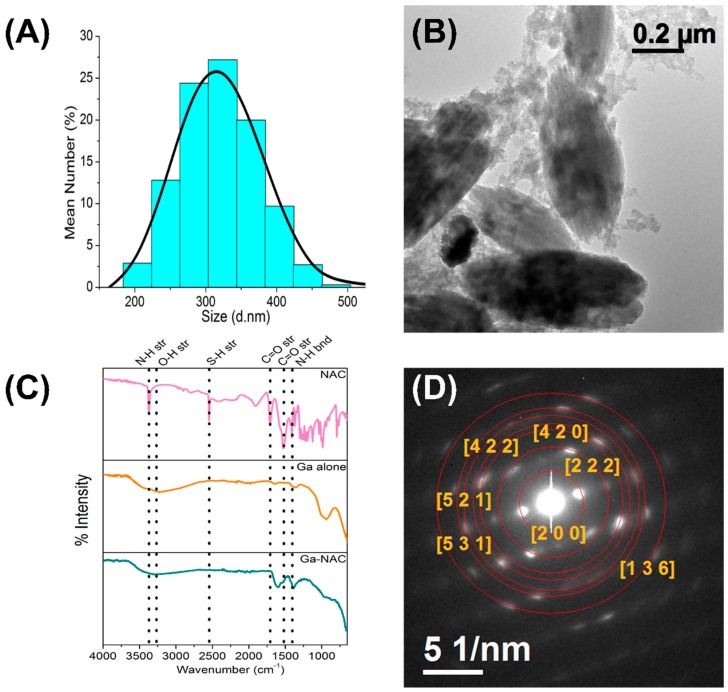
(**A**) Dynamic Light Scattering of NAC-coated Ga particles with the most frequent sizes falling between ~250–~375 nm; (**B**) High-Resolution Transmission Electron Microscopy indicating oval-shaped particles of roughly ~300 nm; (**C**) FTIR analysis of Ga particle indicating NAC interaction; (**D**) SAED analysis from Ga-NAC indicating gallium hydroxide as the major Ga form.

The DLS measurements indicated d ~320 ± 50 nm for the Ga-NAC particles while the TEM images revealed an oval shape (Figure 1A,B). The FTIR fingerprint of the particles (Figure 1C) indicated the presence of a Ga-OH bending in the uncoated gallium particle (943 cm^−1^) and in the Ga-NAC particles (940 cm^−1^). Likewise, O-H stretching was found in uncoated gallium particle (3270 cm^−1^) and Ga-NAC (3327 cm^−1^), as well as N-H bending and C=O stretching (1387 cm^−1^, 1594 cm^−1^ for Ga-NAC) and (1371 cm^−1^, 1534 cm^−1^ for NAC). HR-TEM measurement was carried out to obtain Selected Area Electron Diffraction (SAED) (Figure 1D). Based on the SAED analysis, d-spacing values were calculated for Ga-NAC samples and compared to known Ga-based materials reported in The International Centre for Diffraction Data (ISDD) database. The calculated d-values were inconsistent with known lattice spacing for gallium hydroxide (JCPDS # 01-075-6620) along with the respective (h k l) indexes of (2 0 0), (2 2 2), (4 2 0), (4 2 2), (5 2 1), (5 3 0), (1 3 6).

### 7.2. MIC and MBC

Overall, the MIC and MBC of Ga-NAC (Table 1) and Ga controls ranged between 0.5 and 32 µg/mL. The lowest MIC value of 0.5 µg/mL was obtained with the D-TSB media and Ga-NAC treatment, compared to 1 µg/mL in TSB and 8 µg/mL MHB. In all cases, Ga-NAC particles displayed MIC and MBC values lower than gallium citrate and uncoated gallium particles. In D-TSB, the Ga-NAC treatment performed at similar levels to Colistin.

### 7.3. Cytotoxicity of Ga-NAC Particles

**Figure 2:**
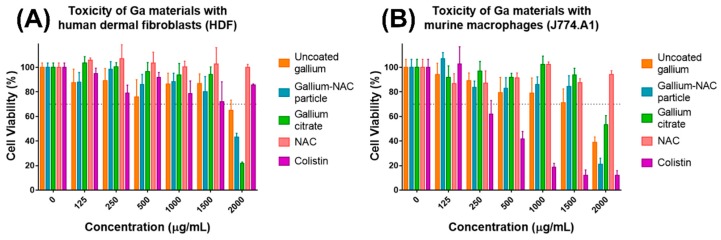
Cytotoxicity assessment of Ga materials. (**A**) Tested with human dermal fibroblasts, all Ga materials exhibited toxicity above 1500 µg/mL. Cells were seeded over a 24 h period before being exposed to antimicrobial for 20–24 h. Colistin did not exhibit any toxicity at the highest concentration tested (2000 µg/mL). (**B**) Tested with murine macrophages, all Ga materials exhibited toxicity above 1500 µg/mL. Colistin was toxic ≥250 µg/mL.

The cell viability of HDF and J774 cells was evaluated using the alamar blue assay. HDF cells treated with Ga materials exhibited toxicity (viability under 70%) above 1500 µg/mL treatment. While they were both toxic above 1500 µg/mL, the cell viability of gallium citrate at 2000 µg/mL was notably lower than the Ga-NAC particle. Colistin and NAC were not toxic at the highest concentrations tested (2000 µg/mL) (Figure 2A). J774 cells treated with Ga materials were toxic above 1500 µg/mL. NAC was not toxic at the highest concentration tested (2000 µg/mL). Colistin displayed toxicity at ≥250 µg/mL.

### 7.4. Biofilm Inhibition and Disruption

**Figure 3:**
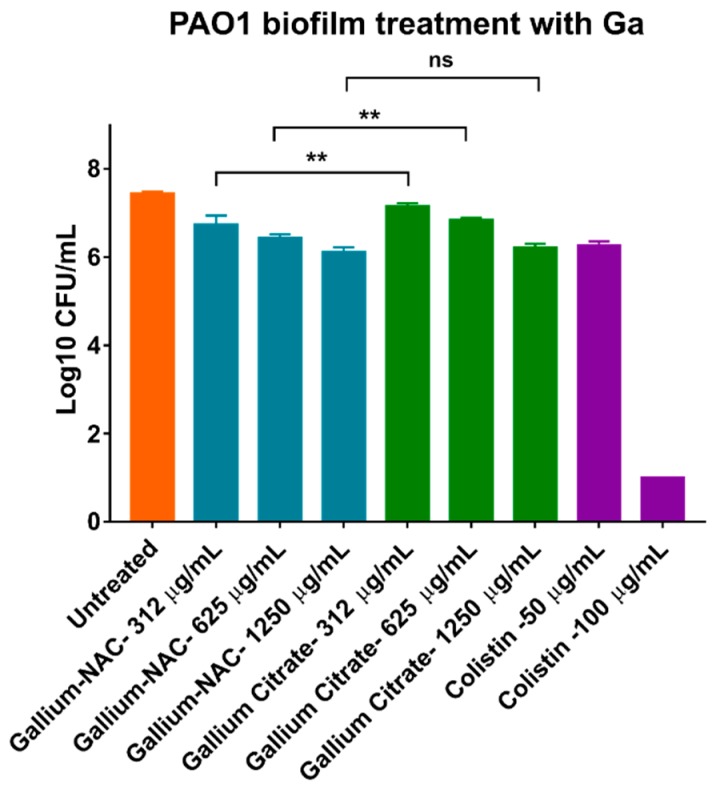
Reduction of viable cells from PAO1 biofilms using Ga treatments. Biofilms were grown for 48 h and treated for 16–20 h at 37 °C as described. Gallium-NAC particle was seen to statistically further reduce viable cells in PAO1 biofilms as compared to Gallium citrate. (ns—Not significant, ** *p* ≤ 0.01). Ga-NAC at 625 µg/mL achieved a 1 log CFU reduction while Ga citrate did not. Colistin achieved a 1 log CFU reduction at 50 µg/mL and completely eradicated the biofilm at 100 µg/mL.

**Figure 4:**
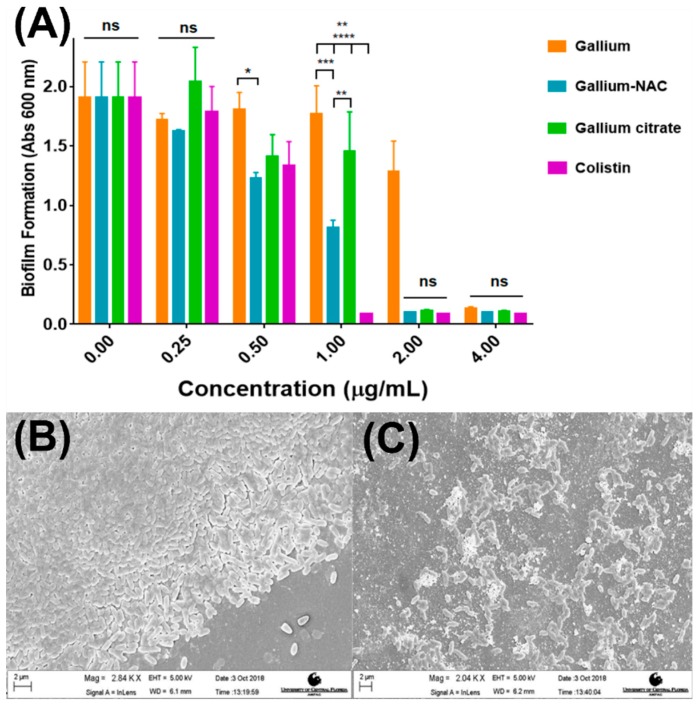
Inhibition and disruption of PAO1 biofilms using Ga treatments. Biofilms were grown on nunc thermanox coverslips for 48 h being treated as described at 37 °C. (**A**) Biofilm formation of PAO1 with Ga treatments. Formation reduced and prevented increasing Ga concentrations. (ns-No significant difference, * *p* ≤0.05, ** *p* ≤ 0.01, *** *p* ≤ 0.001, **** *p* ≤ 0.0001); (**B**) Untreated PAO1 with intact biofilm; (**C**) PAO1 biofilm disrupted and reduced after treatment with 625 µg/mL Ga-NAC particles. Separately conducted CFU analysis indicated 625 µg/mL, which demonstrated a 1 log reduction in the biofilm viability.

Reduction in viable cells within PAO1 biofilms after treatment was assessed using CFU assay. It was seen that all treatments (Colistin, Ga-NAC and Ga citrate) statistically lowered viable cells compared to the untreated control. Colistin was able to completely eradicate PAO1 biofilms cells at 100 µg/mL, while Ga-NAC achieved 1 log reduction at 625 µg/mL, and Ga-citrate achieved 1 log reduction at 1250 µg/mL (Figure 3). The ability of Ga-NAC to prevent biofilm formation was measured using the crystal violet assay. The Ga-NAC was found to inhibit biofilm formation (Figure 4A) above 1 µg/mL, with 1 µg/mL having significantly reduced biofilm formation. At 0.5 µg/mL, all treatments except Ga(OH)_3_ displayed significant reduction compared to the untreated control. At concentrations below 0.5 µg/mL, no significant reduction in biofilm formation was observed compared to the untreated control. At 1 µg/mL, Colistin remained the higher performing treatment with significant improvement over all Ga treatments. However, above 1 µg/mL, Ga-NAC exhibited similar biofilm inhibition to Colistin and higher efficacy than gallium citrate. A control with NAC only demonstrated that NAC alone can inhibit biofilm formation at concentrations over ~2300 ug/mL (Figure S1). At 625 µg/mL metallic Ga, the corresponding NAC concentration was approximately ~2900 ug/mL.

To visualize biofilm disruption, an established biofilm was treated with 625 µg/mL (1 log reduction seen in Figure 3) Ga-NAC particles and imaged using SEM. Untreated PAO1 biofilm (Figure 4B) was full and intact while the treated biofilm (Figure 4C) was disrupted and reduced.

**Figure 5:**
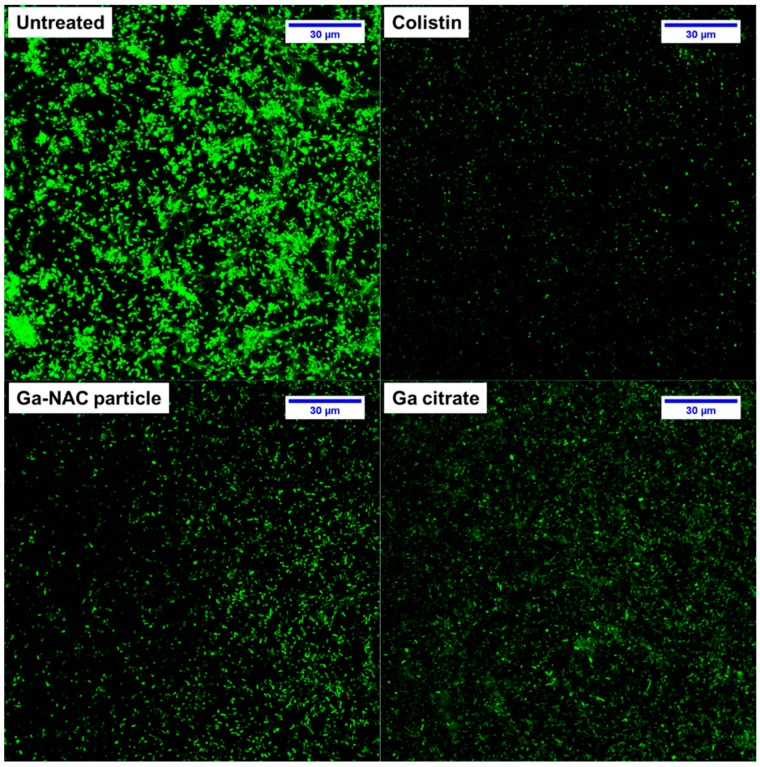
CLSM of gallium-treated *P. aeruginosa* biofilms. *P. aeruginosa* biofilms were formed for 48 h at 37 °C on chambered coverglass slides. After washing, biofilms were treated with Colistin, Ga-NAC or Ga citrate as described and subsequently stained with SYTO 9 for 20 min in the dark. Images were acquired by CLSM using a Leica TCS SP5 100.0X oil objective lens. Representative max stacked views combining all Z-stacks are shown. All treatments displayed a strong visible reduction in cells compared to the untreated control.

**Figure 6:**
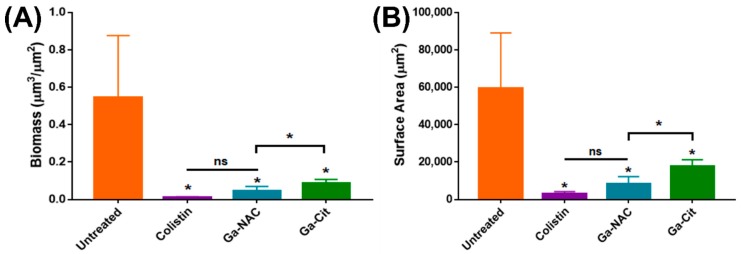
COMSTAT analysis of CLSM images of gallium-treated *P. aeruginosa* biofilms. Ga treatment was conducted at 625 µg/mL and Colistin at 50 µg/mL which obtained a 1 log reduction in bacteria viability, as seen in Figure 4. Biomass (**A**) and surface area (**B**) were quantified from three replicate fields of view in CLSM. (ns—Not significant, * T-Test, *p* < 0.05). All treatments were statistically improved compared to the untreated control. Ga-NAC was statistically better than Ga-Cit while there was no significant difference between Ga-NAC and Colistin.

Based on these results in Figure 3, a CLSM study was conducted on PAO1 biofilms grown on chambered coverglass and treated at 625 µg/mL Ga. Representative max stack images reveal the reduction in biofilm after treatment with 625 µg/mL Ga and 50 µg/mL Colistin (Figure 5). COMSTAT analysis of these images was conducted to determine the biomass and surface after treatment. Ga-NAC was seen to have a statistically lower biomass and surface area compared to Ga citrate (Figure 6A,B).

### 7.5. Cell Membrane Chemistry

**Figure 7:**
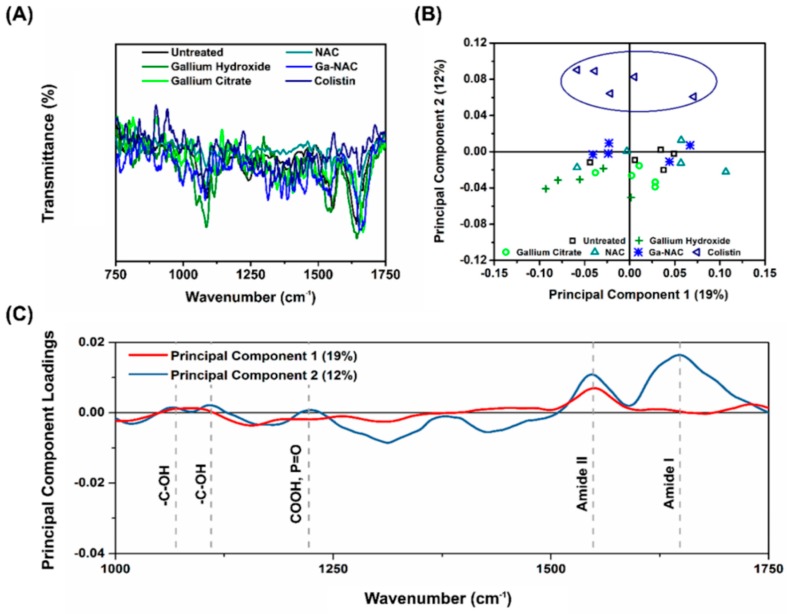
FTIR and PCA analysis of PAO1 treated with Ga formulations. (**A**) Raw FTIR data show no obvious significant shifts or changes to cell membrane chemistry due to treatment, however (**B**) PCA scores plot shows clear separation of cell membrane chemistry variance caused by Colistin alone, and (**C**) PCA loadings shows cause of variance due to a significant change to the Amide I band.

Changes to cell membrane chemistry were evaluated using FTIR (Figure 7A) and PCA analysis (Figure 4B). Colistin was the only treatment with notable changes in IR fingerprint (Figure 7B), as seen with the complete separation of the Colistin treatment in the PCA score plot. The PCA loading curves (Figure 7C) indicated a change in Amide I in the IR fingerprint.

### 7.6. Cell Membrane Degradation Determined by Atomic Force Microscopy (AFM)

**Figure 8:**
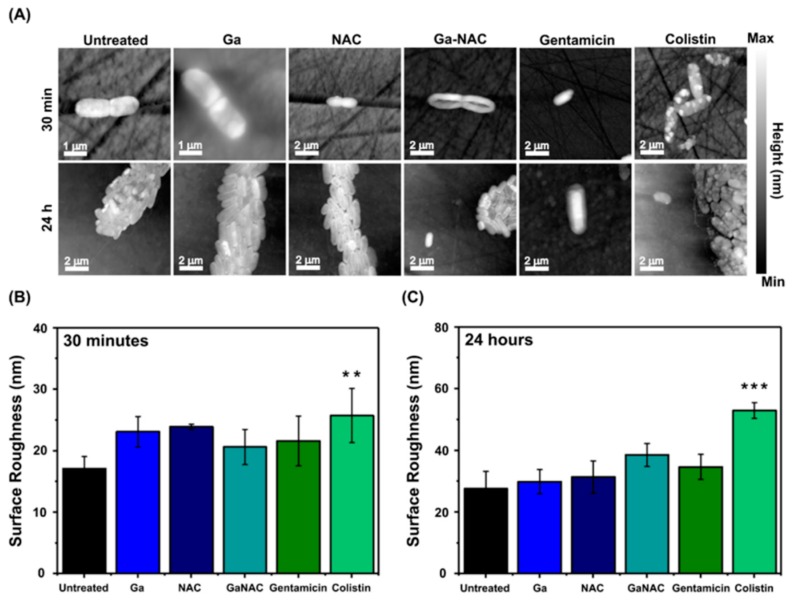
AFM with RMS surface roughness analysis of PAO1 after treatment with Ga formulations. (**A**) Representative AFM height images of bacteria under different treatment conditions at 30 min and 24 h used for measuring RMS surface roughness, (**B**) RMS surface roughness of bacteria after 30 min with the various treatment components showing significant cell membrane roughening with Colistin, and (**C**) RMS surface roughness measurements of treated bacteria after 24 h demonstrating more advanced significant cell membrane roughening by Colistin (** *p* ≤ 0.01, *** *p* ≤ 0.001).

Cell membrane surface roughness was measured using contact mode AFM in ambient, dry conditions. Following a 30 min and 24 h incubation with each treatment, bacteria cells were imaged and RMS surface roughness was determined for each case, as demonstrated by the representative AFM height images (Figure 8A). For both treatment durations, significant shifts in surface roughness were observed only with Colistin, a cell membrane-degrading antibiotic (Figure 8B,C), relative to the untreated control. Notably, however, the Ga-NAC treatment’s surface roughness was similar to that of Gentamicin, a protein-inhibiting antibiotic.

### 7.7. Time Dependent PAO1 Growth Curve with Fe Supplementation

**Figure 9:**
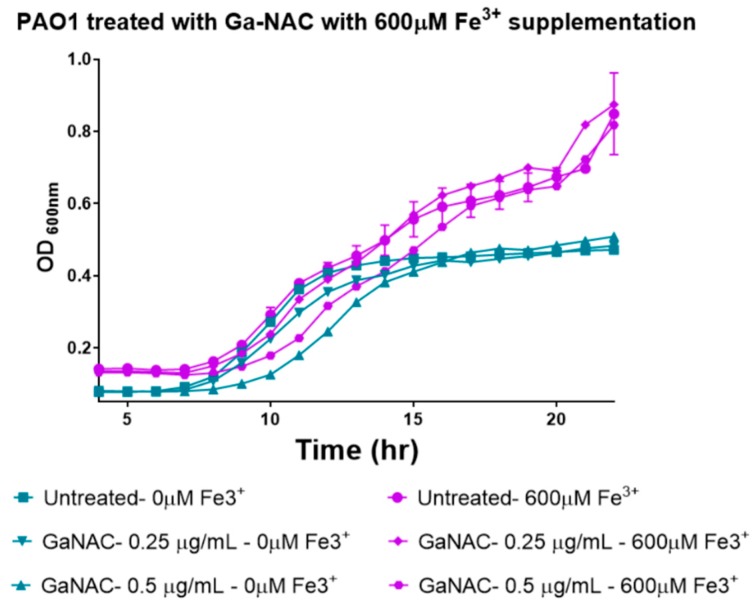
Growth curve of PAO1 with treatment by Ga-NAC particles without Fe supplementation and with Fe supplementation. Reduction in growth seen in the absence of Fe^3+^ was ameliorated with supplementation with Fe^3+^.

PAO1 growth was observed with Ga-NAC treatment in the presence and absence of 600 µM FeCl_3_ supplementation. Ga-NAC without Fe^3+^ treatment significantly reduced PAO1 growth at 0.5 µg/mL and above while the treatment supplemented with Fe^3+^ had a reduced effect with higher PAO1 growth (Figure 9).

### 7.8. Ga release and Association with PAO1

**Figure 10:**
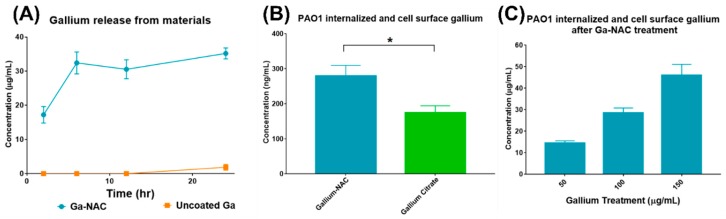
(**A**) Ga release from uncoated and coated particles, (**B**) Ga-cell association with PAO1 after treatment with Ga-NAC particles and Ga citrate; (*) indicates significant difference from paired T-test with *p* < 0.05. (**C**) Dose-dependent Ga-cell association after treatment with Ga-NAC particles.

Ga ions released from coated and uncoated Ga particles were analyzed using a dialysis method. Ga released from NAC-coated Ga particles was much higher than from uncoated particles (Figure 10A). The Ga associated on the PAO1 cell surface and internalized was compared between Ga-NAC particles and Ga citrate after treatment with 10 µg/mL. Ga-NAC had significantly higher Ga association than Ga citrate using a paired T-test with *p* < 0.05 in GraphPad Prism 7 (Figure 10B). The Ga association from Ga-NAC treatment at 50, 100 and 150 µg/mL was analyzed, and a dose-dependent effect was observed (Figure 10C).

## 8. Discussion

In the present study, the antimicrobial efficacy of a novel gallium particle coated with N-acetyl cysteine (Ga-NAC) was assessed to serve as a potential alternative to traditional antibiotics for *Pseudomonas aeruginosa* infections. The gallium particle coated with NAC was synthesized using a sol-gel method and imaged as an oval shape ~320 nm ±50 nm in size (Figure 1A,B). FTIR analysis confirmed that the NAC was successfully attached to the surface of the Ga particle, as specific peaks associated with NAC were found in the spectra of the Ga-NAC sample after washing (Figure 1C SAED analysis indicated that Ga-NAC particles are crystalline in nature (Figure 1D), corresponding to known Gallium Hydroxide (JCPDS # 01-075-6620). MIC and MBC studies (Table 1) on planktonic PAO1 confirmed the potent antimicrobial properties of gallium against PAO1. MIC values of Ga-NAC particles were 1 µg/mL, compared to 2 µg/mL for gallium citrate and 0.5 µg/mL for Colistin when conducted in TSB. The same trend was observed in MHB with slightly higher numbers of 4 µg/mL for Ga-NAC particles and 8 µg/mL for Ga citrate along with 1 µg/mL for Colistin. These results indicate that Ga-NAC activity is similar to traditional anti-pseudonomal antibiotics. Increased activity and lower MIC values were seen in deferrated TSB (D-TSB), which confirmed that the presence of Fe^3+^ lowers Ga activity.

The prepared Ga-NAC particles are proposed for use in *Pseudomonas aeruginosa* skin infections; therefore, the cytotoxicity was assessed using human dermal fibroblasts and murine macrophages. The toxicity of Ga-NAC particles for mammalian cells was low. Concentrations as high as 1500 µg/mL did not reduce cell viability lower than the 70% threshold as referred to in ISO 10993-5:2009 (Figure 2). This, along with the MIC of 1 µg/mL in TSB, indicates a very large therapeutic window (>1000 times MIC) for the Ga-NAC particles. NAC did not exhibit any toxicity at the tested concentrations. Colistin was not observed to damage dermal fibroblast at the tested concentrations; however, it was significantly more toxic than Ga with macrophages having toxicity ≥250 µg/mL, indicating that the therapeutic window (<500 times MIC) for Colistin is much lower than that of Ga materials. Gallium citrate was more toxic since it reduced cell viability more than Ga-NAC. While it was more toxic, gallium citrate is still considered to be safe with previous studies indicating continuous intravenous infusion (200 mg/m^2^/day during 5 days, 500 mg of gallium nitrate (20 mL, 25 mg/mL)) is generally well tolerated [34,35].

The effect of the Ga-NAC particles on preventing biofilm formation or disrupting established biofilms was assessed. The ability of *Pseudomonas aeruginosa* to form biofilms increases its antimicrobial tolerance and makes treating infections including skin infections more challenging. N-acetyl cysteine has been shown in literature to disrupt or prevent biofilm formation suggesting it as an appropriate biocompatible agent to work together with Ga^3+^ against PAO1 biofilms. Beyond biofilm disruption, it was seen in a CFU assay that Ga-NAC treatment can lower the viable cells within PAO1 biofilms after treatment (Figure 3). Ga-NAC was able to lower viable cells statistically more than Ga citrate, and a 1 log reduction was observed at 625 µg/mL with Ga-NAC while it was not seen with Ga citrate until 1250 µg/mL. Colistin was shown to produce a 1 log reduction in bacteria viability at 50 µg/mL and completely eradicated the biofilm at 100 µg/mL. The crystal violet assay conducted to ascertain the potency of Ga at inhibiting biofilm formation indicated that all treatments were effective at preventing PAO1 biofilm formation at 4 µg/mL and above (Figure 4A). The Ga-NAC particles completely prevented biofilm formation starting at 2 µg/mL and drastically reduced formation at 1 µg/mL. This range was comparable to Colistin, which completely prevented biofilm formation at 1 µg/mL and above. The treatment of an established biofilm with 625 µg/mL Ga-NAC disrupted and reduced the biofilm (Figure 4C). This concentration was used as it was shown in Figure 3 to result in a 1 log reduction in bacteria viability in the biofilm. The improved effect of Ga-NAC treatment on established biofilms was additionally studied using CLSM. CLSM image visibility displayed a strong reduction in the numbers of cells seen after treatment with Ga at 625 µg/mL and Colistin at 50 µg/mL (Figure 5). Using COMSTAT analysis software to analyze CLSM images, it was seen that Ga-NAC was able to lower biofilm biomass and surface area more significantly than Ga citrate while being comparable to Colistin (Figure 6A,B). N-acetyl cysteine’s ability to soften biofilms may be attributed to this improved activity. At 625 µg/mL Ga-NAC, the NAC concentration was over ~2900 µg/mL, which was above the concentration shown to inhibit biofilm formation in Figure S1. While Colistin was able to reduce and eradicate the PAO1 biofilm more efficiently than Ga treatments, it was more toxic to murine macrophages (Figure 3). Ga-NAC was able to achieve a comparable MIC to Colistin (1 µg/mL to 0.5 µg/mL).

Overall, the FTIR fingerprints combined with PCA (Figure 7) suggested little to no change to the bacterial cell membrane as a result of the Ga-based treatment, whereas changes, particularly in the Amide I band region, were identified in the bacteria treated with Colistin. To further corroborate these results, we explored more specifically the physical characteristics of the bacteria surface as a function of treatment by using AFM. Following 30 min of treatment, shown in Figure 8B, the untreated control exhibited an average RMS surface roughness of approximately 17 nm (SD ± 2 nm), while gallium hydroxide, NAC, and Ga-NAC were 23, 22, and 21 nm (SD ± 2, 4, 3 nm), respectively. This was analogous to the protein inhibiting antibiotic, Gentamicin, which had an average surface roughness of 31 ± 7 nm. However, Colistin-treated cells showed significantly rougher surfaces at 26 nm (*p* < 0.01). At a longer treatment time (24 h), the untreated control had an average surface roughness of approximately 28 nm (SD ± 6 nm), while the roughness for the gallium hydroxide, NAC, and Ga-NAC treated bacteria were 30, 31, and 39 nm (SD ± 4, 5, and 4 nm), respectively (Figure 8C). Again, Ga-NAC demonstrated a similar average surface roughness to that of Gentamicin, which had an average surface roughness of 34.6 ± 4 nm. Only the change in surface roughness for Colistin-treated cells was significant after 24 h (*p* < 0.001) with a roughness of 53 nm (SD ± 3 nm). Together, the findings suggest that the mode of action of the Ga-NAC treatment was not by cell membrane degradation, but instead the internalization of Ga ions for protein inhibition and/or ROS generation.

To investigate why Ga-NAC particles demonstrated enhanced activity while not causing significant membrane damage, the antimicrobial effect in the presence of Fe^3+^ was tested (Figure 9). It was seen that in the presence of Fe^3+^, the antimicrobial effect of Ga was diminished. This reduction in activity confirms the ability of Ga to perturb metabolism by disrupting Fe-dependent processes. The disruption of Fe-dependent metabolism has been previously indicated as a major part of Ga^3+^-associated toxicity [5]. Metabolic disruption is dependent on Ga^3+^ interacting with bacterial siderophores or the cell surface and being taken up into the cell to bind to metabolic molecules. The Ga particle coated with NAC was more reactive and released a significantly larger amount of Ga^3+^ than the uncoated particle (Figure 10A). When compared to gallium citrate, there was a significantly higher amount of Ga associated with the PAO1 cells, indicating that the enhanced activity demonstrated from the Ga-NAC particle was in part due to the increased release and uptake of Ga (Figure 10B,C). Higher release and uptake of Ga into PAO1 from Ga-NAC indicates that there is a higher bioavailability of Ga from Ga-NAC as compared to Ga citrate.

In the present study, we have demonstrated that a Ga particle coated with NAC offers strong antibacterial and anti-biofilm properties while exhibiting low toxicity, thus possessing a large therapeutic window. The enhanced activity was found to not derive from membrane damage. The mode of action was likely, at least in part, due to increased Ga^3+^ release and interaction with the bacterial cell resulting in higher bioavailability. Therefore, Ga-NAC particles have a strong potential to serve as an alternative to antibiotics in the treatment of *Pseudomonas aeruginosa* skin infections. Future studies will delve deeper into the molecular mechanisms associated with Ga-NAC toxicity against clinically relevant strains.

## Figures and Tables

**Table 1:** MIC and MBC values for Ga compounds tested with PAO1.

Treatment	TSB		D-TSB		MHB	
	MIC (µg/mL)	MBC (µg/mL)	MIC (µg/mL)	MBC (µg/mL)	MIC (µg/mL)	MBC (µg/mL)
Gallium citrate	2	4	2	2	8	16
Uncoated Ga particle	8	16	4	8	16	32
Ga-NAC particle	1	2	0.5	1	4	8
NAC	2500	5000	2500	5000	5000	5000
Colistin	0.5	0.5	0.5	0.5	1	1

## Supplementary Materials

The following are available online at https://www.mdpi.com/2076-0817/8/3/120/s1, Figure S1: Inhibition of PAO1 biofilms using NAC.

## References

[1] Control CFD and Prevention (2013). Antibiotic Resistance Threats in the United States.

[2] Zayyad H., Eliakim-Raz N., Leibovici L., Paul M. (2017). Revival of old antibiotics: needs, the state of evidence and expectations. Int. J. Antimicrob. Agents.

[3] Falagas M.E., Kasiakou S.K., Saravolatz L.D. (2005). Colistin: the revival of polymyxins for the management of multidrug-resistant gram-negative bacterial infections. Clin. Infect. Dis..

[4] Edwards C.L., Hayes R.L. (1969). Tumor scanning with ^67^Ga citrate. J. Nucl. Med..

[5] Kaneko Y., Thoendel M., Olakanmi O., Britigan B.E., Singh P.K. (2007). The transition metal gallium disrupts Pseudomonas aeruginosa iron metabolism and has antimicrobial and antibiofilm activity. J. Clin. Investig..

[6] Halwani M., Yebio B., Suntres Z.E., Alipour M., Azghani A.O., Omri A. (2008). Co-encapsulation of gallium with gentamicin in liposomes enhances antimicrobial activity of gentamicin against Pseudomonas aeruginosa. J. Antimicrob. Chemother..

[7] Richter K., Ramezanpour M., Thomas N., Prestidge C.A., Wormald P.J., Vreugde S. (2016). Mind De GaPP: In vitro efficacy of deferiprone and gallium—Protoporphyrin against Staphylococcus aureus biofilms. Int. Forum Allergy Rhinol..

[8] Rzhepishevska O., Ekstrand-Hammarström B., Popp M., Björn E., Bucht A., Sjöstedt A., Antti H., Ramstedt M. (2011). The antibacterial activity of Ga3+ is influenced by ligand complexation as well as the bacterial carbon source. Antimicrob. Agents Chemother..

[9] Hijazi S., Visaggio D., Pirolo M., Frangipani E., Bernstein L., Visca P. (2018). Antimicrobial activity of gallium compounds on ESKAPE pathogens. Front. Cell. Infect. Microbiol..

[10] DeLeon K., Balldin F., Watters C., Hamood A., Griswold J., Sreedharan S., Rumbaugh K.P. (2009). Gallium maltolate treatment eradicates Pseudomonas aeruginosa infection in thermally injured mice. Antimicrob. Agents Chemother..

[11] Goss C.H., Kaneko Y., Khuu L., Anderson G.D., Ravishankar S., Aitken M.L., Lechtzin N., Zhou G., Czyz D.M., McLean K. (2018). Gallium disrupts bacterial iron metabolism and has therapeutic effects in mice and humans with lung infections. Sci. Transl. Med..

[12] Richter K., Thomas N., Claeys J., McGuane J., Prestidge C.A., Coenye T., Wormald P.J., Vreugde S. (2017). A topical hydrogel with deferiprone and gallium-protoporphyrin targets bacterial iron metabolism and has antibiofilm activity. Antimicrob. Agents Chemother..

[13] Arivett B.A., Fiester S.E., Ohneck E.J., Penwell W.F., Kaufman C.M., Relich R.F., Actis L.A. (2015). Antimicrobial activity of gallium protoporphyrin IX against Acinetobacter baumannii strains displaying different antibiotic resistance phenotypes. Antimicrob. Agents Chemother..

[14] Ooi M.L., Richter K., Drilling A.J., Thomas N., Prestidge C.A., James C., Moratti S., Vreugde S., Psaltis A.J., Wormald P.J. (2018). Safety and Efficacy of Topical Chitogel-Deferiprone-Gallium Protoporphyrin in Sheep Model. Front. Microbiol..

[15] Minandri F., Bonchi C., Frangipani E., Imperi F., Visca P. (2014). Promises and failures of gallium as an antibacterial agent. Future Microbiol..

[16] Oglesby-Sherrouse A.G., Djapgne L., Nguyen A.T., Vasil A.I., Vasil M.L. (2014). The complex interplay of iron, biofilm formation, and mucoidy affecting antimicrobial resistance of Pseudomonas aeruginosa. Pathog. Dis..

[17] Frangipani E., Bonchi C., Minandri F., Imperi F., Visca P. (2014). Pyochelin potentiates the inhibitory activity of gallium on Pseudomonas aeruginosa. Antimicrob. Agents Chemother..

[18] Bonchi C., Frangipani E., Imperi F., Visca P. (2015). Pyoverdine and proteases affect the response of Pseudomonas aeruginosa to gallium in human serum. Antimicrob. Agents Chemother..

[19] Zhao T., Liu Y. (2010). N-acetylcysteine inhibit biofilms produced by *Pseudomonas aeruginosa*. BMC Microbiol..

[20] Suk J.S., Boylan N.J., Trehan K., Tang B.C., Schneider C.S., Lin J.M., Boyle M.P., Zeitlin P.L., Lai S.K., Cooper M.J. (2011). *N*-acetylcysteine enhances cystic fibrosis sputum penetration and airway gene transfer by highly compacted DNA nanoparticles. Mol. Ther..

[21] Dinicola S., De Grazia S., Carlomagno G., Pintucci J.P. (2014). *N*-acetylcysteine as powerful molecule to destroy bacterial biofilms. A systematic review. Eur. Rev. Med. Pharmacol. Sci..

[22] Costa F., Sousa D.M., Parreira P., Lamghari M., Gomes P., Martins M.C.L. (2017). *N*-acetylcysteine-functionalized coating avoids bacterial adhesion and biofilm formation. Sci. Rep..

[23] Liu D., Li J., Pan H., He F., Liu Z., Wu Q., Bai C., Yu S., Yang X. (2016). Potential advantages of a novel chitosan-*N*-acetylcysteine surface modified nanostructured lipid carrier on the performance of ophthalmic delivery of curcumin. Sci. Rep..

[24] Clinical and Laboratory Standards Institute (CLSI) (2012). Methods for Dilution Antimicrobial Susceptibility Tests for Bacteria That Grow Aerobically; Approved Standard-Ninth Edition, in CLSI Document M07-A9.

[25] Visca P., Ciervo A., Sanfilippo V., Orsi N. (1993). Iron-regulated salicylate synthesis by *Pseudomonas* spp.. Microbiology.

[26] Hackel M.A., Tsuji M., Yamano Y., Echols R., Karlowsky J.A., Sahm D.F. (2018). In vitro activity of the siderophore cephalosporin, cefiderocol, against carbapenem-nonsusceptible and multidrug-resistant isolates of Gram-negative bacilli collected worldwide in 2014 to 2016. Antimicro. Agents Chemother..

[27] Standardization I.O.F. (2009). Biological Evaluation of Medical Devices in Part 5: Tests for in Vitro Cytotoxicity.

[28] Nguyen U.T., Wenderska I.B., Chong M.A., Koteva K., Wright G.D., Burrows L.L. (2012). Small-molecule modulators of Listeria monocytogenes biofilm development. Appl. Environ. Microbiol..

[29] Harrison J.J., Turner R.J., Ceri H. (2005). High-throughput metal susceptibility testing of microbial biofilms. BMC Microbiol..

[30] Chen H., Wubbolts R.W., Haagsman H.P., Veldhuizen E.J.A. (2018). Inhibition and eradication of *Pseudomonas aeruginosa* biofilms by host defence peptides. Sci. Rep..

[31] Heydorn A., Nielsen A.T., Hentzer M., Sternberg C., Givskov M., Ersbøll B.K., Molin S. (2000). Quantification of biofilm structures by the novel computer program COMSTAT. Microbiology.

[32] Vorregaard M. (2008). Comstat2-a Modern 3D Image Analysis Environment for Biofilms.

[33] Nečas DKlapetek P. (2012). Gwyddion: An open-source software for SPM data analysis. Open Phys..

[34] Smani Y., Canturri A.M., Algaba R.A. (2019). Drug repurposing for the treatment of bacterial and fungal infections. Front. Microbiol..

[35] Warrell R.P., Israel R., Frisone M., Snyder T., Gaynor J.J., Bockman R.S. (1988). Gallium nitrate for acute treatment of cancer-related hypercalcemia: A randomized, double-blind comparison to calcitonin. Ann. Intern. Med..

