# Supplementary material for: N-acetyl Cysteine Coated Gallium Particles Demonstrate High Potency against Pseudomonas aeruginosa PAO1
Source: Pathogens. 2019 Aug 1;8(3):120. doi: 10.3390/pathogens8030120 (PMC6789799; doi:10.3390/pathogens8030120)
Supplement: Supplementary file 1 [file pathogens-08-00120-s001.pdf]

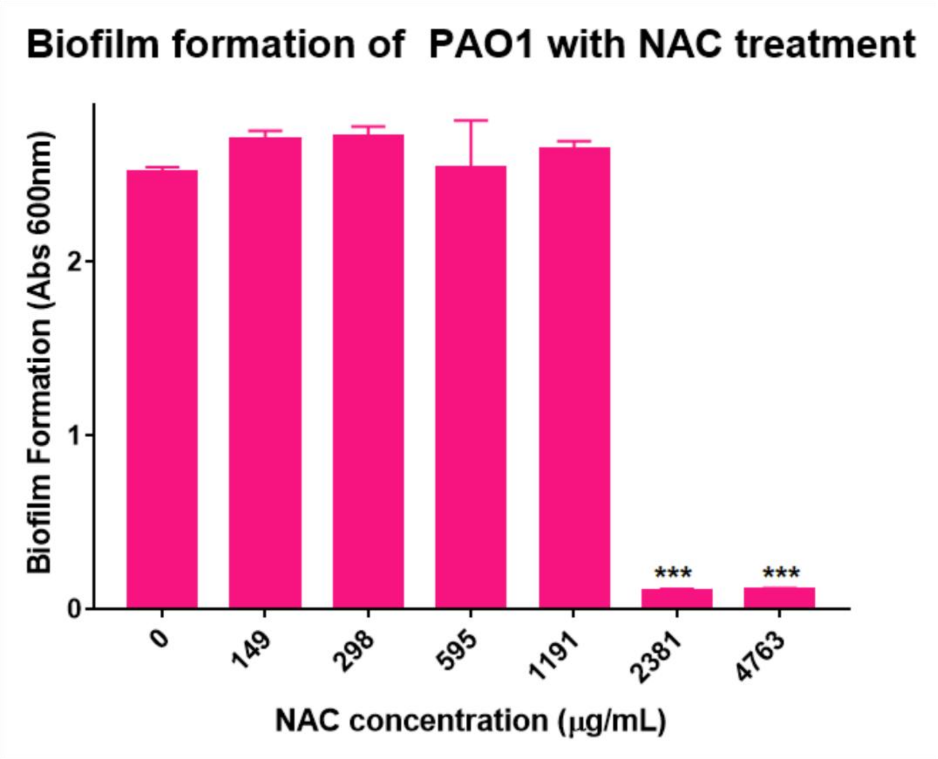

Supplementary Figure 1. Inhibition of PAO1 biofilms using NAC. Biofilms were grown in nunc TSP plate and lids for 24 hrs in the presence of NAC at 37°C. Biofilm formation reduced and prevented with increasing NAC concentrations. (***$p \leq 0.001$ compared to 0 μg/mL concentration, Paired T-test).
